# On the benefits of representation regularization in invariance based domain generalization

**DOI:** 10.1007/s10994-021-06080-w

**Published:** 2022-01-01

**Authors:** Changjian Shui, Boyu Wang, Christian Gagné

**Affiliations:** 1 Mila, Université Laval, Quebec City, G1V 0A6 Canada; 2 Vector Institute, Western University, London, N6A 5B7 Canada; 3 Canada CIFAR AI Chair, Mila, Université Laval, Quebec City, G1V 0A6 Canada

**Keywords:** Domain generalization, Transfer learning, Representation learning

## Abstract

A crucial aspect of reliable machine learning is to design a deployable system for generalizing new related but unobserved environments. Domain generalization aims to alleviate such a prediction gap between the observed and unseen environments. Previous approaches commonly incorporated learning the invariant representation for achieving good empirical performance. In this paper, we reveal that merely learning the invariant representation is vulnerable to the related unseen environment. To this end, we derive a novel theoretical analysis to control the unseen test environment error in the representation learning, which highlights the importance of controlling the smoothness of representation. In practice, our analysis further inspires an efficient regularization method to improve the robustness in domain generalization. The proposed regularization is orthogonal to and can be straightforwardly adopted in existing domain generalization algorithms that ensure invariant representation learning. Empirical results show that our algorithm outperforms the base versions in various datasets and invariance criteria.

## Introduction

Most research in deep learning assumes that models are trained and tested from a fixed distribution. However, such deep models generally fail to adopt in real-world applications, because the test environment is often different from the training (or observed) distributions. Thus, the capacity in generalizing the new environment with a small prediction error is crucial for developing reliable and deployable deep learning systems (Goodfellow et al. 2014). For instance, in autonomous driving, the decision-making system is trained in several specific regions. However, the prediction performance can dramatically degrade in other regions with the same object but different environmental backgrounds.

To this end, *domain generalization* is recently proposed and further analyzed to alleviate the prediction gap between the observed training (${\mathcal {S}}$) and *unseen* test (${\mathcal {T}}$) dataset. Taking the advantage of the shared knowledge (or inductive bias) from multiple observed sources, the prediction on the test environment can be guaranteed (Baxter 2000).

Meanwhile, extrapolation to a new environment is often challenging since the distribution shifts between the training and test environment are inevitable and unknown in advance. Such changes typically include the covariate shift (i.e, the marginal distributions w.r.t. *x* are different ${\mathcal {S}}(x)\ne {\mathcal {T}}(x)$) (Sugiyama et al. 2007), conditional shift (different decision boundaries with ${\mathcal {S}}(y|x)\ne {\mathcal {T}}(y|x)$) (Li et al. 2018; Arjovsky et al. 2019) or both. Based on different distribution-shift assumptions, a widely adopted principle is to learn the representation to satisfy several invariance criteria (Bühlmann 2020) among the observed environments (i.e, sources ${\mathcal {S}}$). Through minimizing the source prediction risk and enforcing the invariance, the prediction performance can be improved (Matsuura and Harada 2020; Li et al. 2018).

Although the idea of learning invariance is quite popular in domain generalization with practical success, several theoretical questions remain elusive. For instance, *is it sufficient to merely learn an invariant representation and minimize source risks to guarantee a good performance in a new related environment? What are the sufficient conditions to guarantee a small test environment error?*

*Contributions* In this paper, we aim to address these theoretical problems in the representation learning-based domain generalization. Concretely, (1) we reveal the limitation of representation learning in domain generalization through barely ensuring invariance criteria, which can lead to a *over-matching* on the observed environments. i.e: the complex or non-smooth representation function can even be vulnerable to the small distribution shift. (2) We derive novel theoretical analysis to upper bound the unseen test environment error in the context of representation learning, which highlights the importance of controlling the complexity of the representation function. We then further formally demonstrate the Lipschitz property as one sufficient condition to ensure the smoothness of the representation. (3) In practice, we propose the *Jacobian matrix regularization* of the representation as a new criterion. The empirical results in various invariance criteria and datasets suggest a consistent improved performance in the test environment.

## Background and motivation

Throughout this paper, we have *T* observed (source) environments ${\mathcal {S}}_1(x,y) ,\dots ,{\mathcal {S}}_T(x,y)$ with $x\in {\mathcal {X}}$, $y\in {\mathcal {Y}}$. The goal of domain generalization is to learn a proper representation function $\phi :{\mathcal {X}}\rightarrow {\mathcal {Z}}$ and classifier $h:{\mathcal {Z}}\rightarrow {\mathcal {Y}}$ to have a good performance on a (unseen) but related test environment ${\mathcal {T}}(x,y)$.

Specifically, let ${\mathcal {L}}$ denote the prediction loss function, then domain generalization can be generally formulated as minimizing the following loss w.r.t. $(\phi ,h)$:

1 $$\begin{aligned} \min _{\phi ,h} \sum _{t} {\mathbb {E}}_{(x,y)\sim {\mathcal {S}}_t} {\mathcal {L}}(h \circ \phi (x), y) + \lambda _0\, \text {INV}(\phi ,{\mathcal {S}}_1,\dots ,{\mathcal {S}}_T) \end{aligned}$$

where $\text {INV}(\phi ,{\mathcal {S}}_1,\dots ,{\mathcal {S}}_T)$ is an auxiliary task to ensure the invariance among the observable source environments, which can have various forms:

*Marginal feature invariance* Ganin et al. (2016) aims at enforcing

$$\begin{aligned} {\mathbb {E}}_{x_1\sim {\mathcal {S}}_1(x)}[\phi (x_1)] = {\mathbb {E}}_{x_t\sim {\mathcal {S}}_2(x)}[\phi (x_2)] = \dots = {\mathbb {E}}_{x_T\sim {\mathcal {S}}_T(x)}[\phi (x_T)], \forall t\in \{1,\dots ,T\}. \end{aligned}$$

Intuitively, the marginal feature invariance encourages all environments shared the same marginal distribution w.r.t. *z*.

*Feature conditional invariance* Zhang et al. (2013) aims at enforcing

$$\begin{aligned} {\mathbb {E}}_{x_1\sim {\mathcal {S}}_1(x|Y=y)}[\phi (x_1)|y] = {\mathbb {E}}_{x_2\sim {\mathcal {S}}_2(x|Y=y)}[\phi (x_2)|y] = \dots = {\mathbb {E}}_{x_T\sim {\mathcal {S}}_T(x|Y=y)}[\phi (x_T)|y], \end{aligned}$$

for $\forall , t, y$. Intuitively, the feature conditional invariance encourages the same distribution of *z*, given the label $Y=y$.

*Label conditional invariance* Arjovsky et al. (2019) and Kamath et al. (2021) aims at enforcing

$$\begin{aligned} {\mathbb {E}}_{x_1\sim {\mathcal {S}}_1(x)}[y|\phi (x_1)=z] = {\mathbb {E}}_{x_2\sim {\mathcal {S}}_2(x)}[y|\phi (x_2)=z] = \dots = {\mathbb {E}}_{x_T\sim {\mathcal {S}}_T(x)}[y|\phi (x_T)=z], \end{aligned}$$

with $\forall t, y$. Intuitively, the label conditional invariance encourages the same decision boundary ${\mathbb {P}}(y|z)$.

The aforementioned invariance principle and its variants have been widely applied in domain generalization with various empirical algorithms. We will show that merely optimizing Eq. (1) with different invariance criteria can be insufficient to guarantee a small prediction error in the related test environment.

**Fig. 1:**
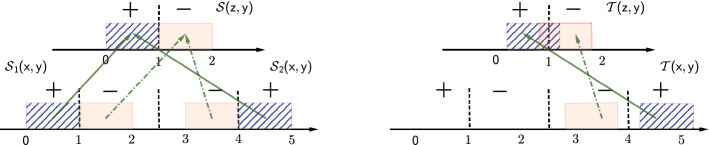
Limitations of optimizing Eq. (1) with conditional invariance criteria. The conditional invariance learns an *over-matched* representation on the training environments (left), which can induce the non-ignorable prediction error in the related test environment (right)

*Limitation of Learning Marginal Invariance* Simultaneously enforcing marginal invariance and minimizing prediction risk have been proved problematic when label distributions (${\mathbb {P}}(y)$) are different (Li et al. 2018). For instance, consider only one source environment ${\mathcal {S}}(x,y)$ and testing environment *T*(*x*, *y*) with binary classification, where the only difference between two environments lies in different label distributions ${\mathcal {S}}(y=1)=0.1$ and ${\mathcal {T}}(y=1)=0.9$. We further suppose there is an embedding $\phi$ and classifier *h* such that ${\mathcal {S}}(\phi (x))={\mathcal {T}}(\phi (x))$ and $R_{{\mathcal {S}}}(h,\phi )=0$. Then it will enforce the ${\mathcal {S}}({\hat{y}}) = {\mathcal {T}}({\hat{y}})$, where ${\hat{y}} = h\circ \phi (x)$. Based on this, the test prediction error will be at most 0.2, despite the identical marginal distribution and zero source risk.

*Limitation of Learning Conditional Invariance* Compared to marginal invariance, feature and label conditional invariance impose stronger principles. However, the prediction can be still vulnerable in the related test environment due to the *over-matching*. Specifically, in Fig. 1, if we adopt the embedding function $\phi$ and classifier *h* as:

$$\begin{aligned} \phi (x) = {\left\{ \begin{array}{ll} x &{} 0 \le x \le 2 \\ x-2 &{} 3\le x\le 4 \\ 5-x &{} 4 < x \le 5 \end{array}\right. }, \quad \quad h(z) = -{\mathrm {sign}}(z-1). \end{aligned}$$

In the latent space *z*, $\forall y \in {\mathcal {Y}}$ we have the conditional invariance with ${\mathcal {S}}_1(y|z)={\mathcal {S}}_2(y|z)$ and ${\mathcal {S}}_1(z|y)={\mathcal {S}}_2(z|y)$ and zero prediction error in the observed environments with ${\mathbb {E}}_{(x,y)\sim {\mathcal {S}}_{t}}{\mathcal {L}}(h\circ \phi (x),y) = 0$. However, in the test time, the unseen environment has a *consistent* distribution shift in Fig. 1(b, Right) such that $\forall y$, $d_{{\mathrm {TV}}}({\mathcal {T}}(x|Y=y)\Vert {\mathcal {S}}_2(x|Y=y))=\epsilon$ with $0<\epsilon <0.5$, then the prediction error w.r.t. (0-1) binary loss is ${\mathbb {E}}_{(x,y)\sim {\mathcal {T}}}{\mathcal {L}}(h\circ \phi (x),y) = \epsilon$, which is vulnerable and non-ignorable in the consistent distribution shift. Moreover, this problem can be much more severe in high-dimensional dataset and over-parametrized deep neural networks.

The problem comes from the *over-matching* of the embedding function, where there exist infinite $\phi$ to minimize Eq. (1) in Fig. 1. However, some embedding functions are rather complex, which are poorly generalized to the related environment. In fact, only a subset of $\phi$ are more robust for the consist environment shift, which suggests a proper model selection w.r.t. $\phi$:

2 $$\begin{aligned} \min _{\phi ,h} \sum _{t} {\mathbb {E}}_{(x,y)\sim {\mathcal {S}}_t} {\mathcal {L}}(h \circ \phi (x), y) + \lambda _0\, \text {INV}(\phi ,{\mathcal {S}}_1,\dots ,{\mathcal {S}}_T) + \lambda _1 {\text {Model}\_\text {Select}(\phi )}. \end{aligned}$$

In the following sections, we will derive theoretical results to demonstrate the influence of model selection w.r.t. $\phi$.

## Theoretical analysis

We aim at proposing a formal understanding of the regularization term in predicting the unseen test environment. We assume the embedding as a random transformation (or transition probability kernel) ${\varPhi }(z|x):{\mathcal {X}}\rightarrow {\mathcal {Z}}$. Then the deterministic representation function is a special case with ${\varPhi }(z|X=x) = \delta _{\phi (x)}$, where $\delta$ is the delta dirac function. The conditional distribution defined on the latent space ${\mathcal {Z}}$ is denoted as ${\mathcal {S}}(z) = \int {\varPhi }(z|x){\mathcal {S}}(x)dx$ and ${\mathcal {S}}(z|Y=y) = \int {\varPhi }(z|x){\mathcal {S}}(x|Y=y)dx$. Before presenting the theoretical results, we discuss several important notations in our paper.

*Performance Metric* Throughout this paper we use *Balanced Error Rate* (BER) rather than the conventional population loss to measure the performance, because the training and test environments can be highly label imbalanced. Namely, conventional population loss may not properly reflect the performance, through ignoring the fewer-samples catalogs. Therefore, the balanced prediction risk w.r.t. classifier *h* and embedding distribution ${\varPhi }$ is

$$\begin{aligned} \text {BER}_{{\mathcal {D}}}(h,{\varPhi }) = \frac{1}{|{\mathcal {Y}}|}\sum _{y} {\mathbb {E}}_{z\sim {\mathcal {D}}(z|Y=y)} {\mathcal {L}}(h(z),y) \end{aligned}$$

Intuitively, BER measure the uniform-average classification error for each class *y*.

*Invariance Criteria* In our analysis, we mainly focus on the feature conditional invariance ${\mathbb {P}}(z|y)$ since the label information is generally discrete or low-dimensional. Then it is relatively straightforward to realize in practice. We will further justify that the feature conditional invariance can also induce the label-conditional invariance and marginal invariance, shown in Lemma 1.

*Distribution Similarity Metric* Besides, we need to specify the metric to measure the similarity between different distributions. In this paper, we adopt the Total Variation (TV) distance (Lin 1991):

$$\begin{aligned} d_{\text {TV}}({\mathcal {S}}_1(x)\Vert {\mathcal {S}}_2(x)) = \int _{x}|{\mathcal {S}}_1(x)-{\mathcal {S}}_2(x)|dx \end{aligned}$$

It is worth mentioning that Jensen–Shannon divergence is the upper bound of TV distance (Polyanskiy and Wu 2019).

Based on these components, we can demonstrate the risk of test environment in the context of representation learning.

### **Proposition 1**

*Supposing*

(i) *observed source environments are* ${\mathcal {S}}_1(x,y),\dots ,{\mathcal {S}}_T(x,y)$ *and unseen test environment is* ${\mathcal {T}}(x,y);$
(ii) *the prediction loss* ${\mathcal {L}}$ *is bounded in* [0, 1]; 
(iii) *the embedding distribution* ${\varPhi }$ *satisfies a small feature-conditional total variation distance on the* *latent space* ${\mathcal {Z}}:$ $\forall i,j \in \{1,\dots ,T\}\, y\in {\mathcal {Y}},$ $d_{{\mathrm {TV}}}({\mathcal {S}}_i(z|Y=y)\Vert {\mathcal {S}}_j(z|Y=y))\le \kappa ;$
(iv) $\forall y \in {\mathcal {Y}}$, *on the* *raw feature space* ${\mathcal {X}}:$ $\underset{t\in \{1,\dots ,T\}}{\min }\,d_{{\mathrm {TV}}}({\mathcal {T}}(x|Y=y)\Vert {\mathcal {S}}_t(x|Y=y)) \le \epsilon$.

*Then the Balanced Error Rate in the test environment is upper bounded by:*

$$\begin{aligned} \text {BER}_{{\mathcal {T}}}(h,{\varPhi }) \le \frac{1}{T} \sum _{t=1}^T \text {BER}_{{\mathcal {S}}_t}(h,{\varPhi }) + \kappa + \alpha _{{\mathrm {TV}}}({\varPhi })\epsilon \end{aligned}$$

*Where* $\alpha _{{\mathrm {TV}}}({\varPhi })$ *is Dobrushin coefficient* *(*Polyanskiy and Wu 2019*):* $\alpha _{{\mathrm {TV}}}({\varPhi }) :=\sup _{x,x^{\prime }\in {\mathcal {X}}} d_{{\mathrm {TV}}}({\varPhi }(\cdot |x)\Vert {\varPhi }(\cdot |x^{\prime }))$

**Fig. 2:**
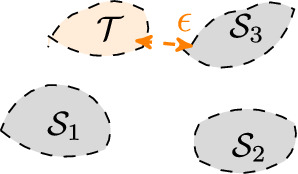
Illustration of $\epsilon$: distance between ${\mathcal {T}}$ and its nearest source ${\mathcal {S}}_3$

*Discussions* The prediction risk of an unseen test environment is controlled by the following terms:

1. The first term suggests to learn *h* and ${\varPhi }$ to minimize the BER over the labeled data from the source environments;
2. A small $\kappa$ indicates learning ${\varPhi }$ to match feature-conditional distribution. Specifically, when $\kappa =0$, we have ${\mathcal {S}}_1(z|Y=y)=\dots ={\mathcal {S}}_T(z|Y=y)$, achieving feature-conditional invariance;
3. $\epsilon$ in the third term is an *unobservable* factor in the learning. As Fig. 2 shows, $\epsilon$ reveals the inherent relations between the test and source environments. Intuitively, a small $\epsilon$ means the test environment ${\mathcal {T}}$ is similar to one of the observed sources, which indicates that we are easier to predict the test environment. If $\epsilon$ is too large, then the source and the test distribution can be indeed quite different, which suggests the generalization to this new environment could be more challenging.
4. $\alpha _{{\mathrm {TV}}}({\varPhi })$ in the third term is a *controllable* factor as a regularization w.r.t. ${\varPhi }$. Intuitively, $\alpha _{{\mathrm {TV}}}({\varPhi })$ reflects the smoothness of the embedding. In the test time, the regularization on ${\varPhi }$ is crucial since the $\epsilon$ is *unknown, uncontrollable and even non-ignorable*. That is, merely minimizing Eq. (1) by ensuring $\text {BER}_{{\mathcal {S}}_t}(h,{\varPhi })=0$ and $\kappa =0$ are not sufficient. If $\alpha _{{\mathrm {TV}}}({\varPhi })$ is relatively large, the upper bound will become vacuous, and generalization in the test environment is not necessarily guaranteed;
5. The trade-off in learning ${\varPhi }$. Although $\alpha _{{\mathrm {TV}}}({\varPhi })$ suggests a regularization term, however over-regularization can be harmful in learning meaningful representations. Consider an extreme scenario, when the embedding distribution ${\varPhi }$ is a constant, then $\alpha _{{\mathrm {TV}}}({\varPhi })=0$, the network does not learn an embedding and $\text {BER}_{{\mathcal {S}}_t}(h,{\varPhi })$ will be inherently large.

Compared with most previous theoretical results, our results highlight the role of representation learning in domain generalization. In particular, Theorem 1 further motivates the new principles to control the Dobrushin Coefficient, which will be illustrated in Sects. 3.2 and 4.

### Relation to other invariance criteria

Proposition 1 verifies the importance of considering regularizing of ${\varPhi }$ under feature-conditional invariance, the following lemma reveals the relations between feature-conditional invariance and other two invariance criteria.

#### **Lemma 1**

*If the embedding distribution* ${\varPhi }$ *satisfies a small feature-conditional total variation distance on the* *latent space* ${\mathcal {Z}}:$ $\forall i,j \in \{1,\dots ,T\}\, y\in {\mathcal {Y}}$, $d_{{\mathrm {TV}}}({\mathcal {S}}_i(z|Y=y)\Vert {\mathcal {S}}_j(z|Y=y))\le \kappa$ *and* ${\mathcal {S}}_i(Y=y)={\mathcal {S}}_j(Y=y)=\frac{1}{|{\mathcal {Y}}|}$*, then we have*

$$\begin{aligned} {\mathbb {E}}_{z\sim {\varOmega }^{\star }} |{\mathcal {S}}_i(y|z)-{\mathcal {S}}_j(y|z)|\le C^{+}\kappa , \quad {\mathbb {E}}_{z\sim {\varOmega }^{\star }} |{\mathcal {S}}_i(z)-{\mathcal {S}}_j(z)|\le \kappa , \end{aligned}$$

*where* $C^{+}$ *is a positive constant and* ${\varOmega }^{\star } = \text {supp}({\mathcal {S}}_i(z))\cap \text {supp}({\mathcal {S}}_j(z))$ *denotes the intersection of latent space between environment* *i* *and* *j*.

Lemma 1 reveals that the feature conditional invariance can induce other types of invariance if the label distribution among the sources is balanced, which is practically feasible through re-sampling the dataset as a uniform distribution. Specifically, if $\kappa =0$, we can achieve the other two invariance criteria.

### Sufficient conditions for controlling Dobrushin coefficient

We have proved the importance of a small Dobrushin Coefficient for guaranteeing the test environment risk. In this section, we will discuss the sufficient conditions that controls the Dobrushin Coefficient. Lemma 2 reveals one sufficient condition: a Lipschitz representation can control $\alpha _{{\mathrm {TV}}}({\varPhi })$.^1^

#### **Lemma 2**

*Supposing the embedding distribution* ${\varPhi }(z|x)$ is *d-dimensional parametric Gaussian distribution with* $z\sim {\mathcal {N}}(\phi (x),\sigma ^2{\mathbf {I}}_{d})$ *and* $d_{\max } = \sup _{x,x^{\prime }\in {\mathcal {X}}}\,\Vert x-x^{\prime }\Vert _2$*, then the Dobrushin Coefficient is upper bounded by:*

$$\begin{aligned} \alpha _{{\mathrm {TV}}}({\varPhi }) \le \sqrt{2}\left( 1- \exp (-\frac{d^2_{\max }}{8d \sigma ^2} L^2_{\phi } )\right) ^{1/2} \end{aligned}$$

*where* $L_{\phi }$ *is the Lipschitz constant of* $\mu _{\phi }(x)$*, i.e* $\forall x,x^{\prime }\in {\mathcal {X}}, \Vert \phi (x)-\phi (x^{\prime })\Vert \le L_{\phi } \Vert x-x^{\prime } \Vert _2$.

In the conventional deep neural-network, the deterministic parametric embedding can be approximated as the mean ($\phi (x)$) of the conditional distribution with a small variance (Achille and Soatto 2018). Therefore, Lemma 2 suggests that learning a Lipschitz embedding can promote a better generalization property in the related test environment ${\mathcal {T}}$.

## Practical implementations

We have demonstrated the Lipschitz property of the embedding function $\phi$ can induce a regularization property, resulting a better generalization. In this section, we will further elaborate practical implementations to realize the Lipschitz property of the embedding function through multiple observed source environments.

**Fig. 3:**
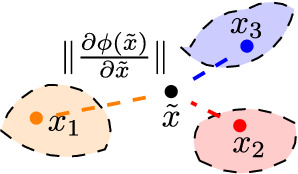
Illustration of the virtual sample generation

It has been proved that the Frobenius norm of Jacobian matrix w.r.t $\phi$ is the upper bound of the Lipschitz constant of $\phi$ (Miyato et al. 2018). In domain generalization, we generally have multiple environments. In this context, the regularization is conducted on the virtual samples ${\tilde{x}}$, which are generated through these environments. Intuitively, the virtual samples can be created outside the support of different environments, shown in Fig. 3. Then conducting a regularization on the virtual samples can effective ensure the Lipschitz property on these *unobserved* regions.

For an efficient generation, we create virtual samples through a linear combination of *x* from each source, shown in Fig. 3. As for determining the linear combination coefficients, we generate the coefficients $(\gamma _1,\dots ,\gamma _T)$ through the Dirichlet distribution with hyper-parameter $\beta =1$, which is inspired from Zhang et al. (2017). The proposed algorithm is presented in Algorithm 1.
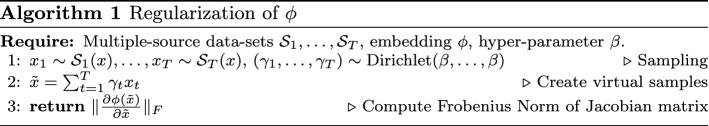


*Regularization is independent of learning invariance* We denote the $\text {INV}(\phi ,{\mathcal {S}}_1,\dots ,{\mathcal {S}}_T)$ as the algorithms that achieve invariance (e.g., marginal, label and feature conditional invariance), which includes a wide range of practical algorithms. Then the improved loss can be expressed as:

$$\begin{aligned} \min _{\phi ,h} \frac{1}{T}\sum _{t}{\mathrm {BER}}_{{\mathcal {S}}_t}(h\circ \phi ) + \lambda _0\, \text {INV}(\phi ,{\mathcal {S}}_1,\dots ,{\mathcal {S}}_T) + \lambda _1 {\mathbb {E}}_{{\tilde{x}}} \Vert \frac{\partial \phi ({\tilde{x}})}{\partial {\tilde{x}}}\Vert _{F}. \end{aligned}$$

In the experimental part, we will investigate different invariance principles and demonstrate the benefits of our regularization.

## Related work

*Learning invariance* is a popular and widely adopted principle in domain generalization. Inspired from the techniques in deep domain adaptation (Ben-David et al. 2010), enormous approaches have been proposed to enable different invariance criteria such as marginal invariance ${\mathcal {S}}_1(z)=\dots ={\mathcal {S}}_T(z)$ (Ganin et al. 2016; Li et al. 2018; Sicilia et al. 2021; Albuquerque et al. 2019). However, the proposed theoretical results are mainly inspired from unsupervised domain adaptation, which does not consider the specific scenarios in domain generalization. i.e, the label information is known during the source alignment, which can induce better matching approaches. As for feature conditional invariance ${\mathcal {S}}_1(z|y)=\dots ={\mathcal {S}}_T(z|y)$ (Li et al. 2018; Wang et al. 2020; Zhao et al. 2020; Ilse et al. 2019), it considers the label information and enforces stronger conditions among the sources. However, as our counterexample indicates, merely learning the conditional invariance can be insufficient to guarantee the unseen test prediction risk. In contrast, we further formally reveal the limitation of representation learning w.r.t. conditional invariance, which remains elusive in the previous work.

A more recent approach is to learn label conditional invariance, i.e., ensuring the same decision boundary across different environments (IRM; Arjovsky et al. 2019; Lu et al. 2021). However, recent work reveals the potential failure scenarios in IRM (Kamath et al. 2021), which can be explained from our theoretical analysis.

Another promising direction in domain generalization is to incorporate with meta-learning (Deshmukh et al. 2019; Blanchard et al. 2011), which assumes the training and testing environment are i.i.d. (Independent and identically distributed) sampled from a meta-distribution. Then through learning a good meta-parameter, we have a good prediction performance in the test distribution. However, the challenging lies in the i.i.d. assumption, i.e, the tasks may not be necessarily independently generated such as ColorMNIST. Thus, the meta-learning theory can be restrictive in domain generalization.

*Relation with data-augmentation based Approach* It has been recently observed that data-augmentation based approaches are quite effective in various practical domain generalization (Volpi et al. 2018; Li et al. 2019; Zhou et al. 2020, 2021; Müller et al. 2020). Intuitively, data augmentation aims at generating new samples from observed environments to induce smooth predictions. In this part, we aim to analyze the role of data-augmentation, which is *implicit* to learn a Lipschitz representation and consistent with our theoretical results.

Specifically, we consider one typical case with a conditional interpolation function $\text {INP}$ with ${\tilde{x}} = \text {INP}(x_1,\dots ,x_T; y)$ with $x_1\sim {\mathcal {S}}_1(x|y), \dots , x_T\sim {\mathcal {S}}_T(x|y)$. For instance, considering object classification under different background, the conditional augmentation aims at creating the same but new object through considering information from different environments. We further suppose the binary classification problem with ${\mathcal {Y}}=\{-1,+1\}$, the classifier is linear with $h(z)=w^T z$ and the prediction loss is logistic loss with ${\mathcal {L}}({\hat{y}},y)= \log (1+\exp (-{\hat{y}}y))$. The augmentation loss can be written as:

$$\begin{aligned} R_{\text {aug}} = \sum _{y} {\mathbb {E}}_{{\tilde{x}}\sim \text {INP}(x_1,\dots ,x_T; y)} {\mathcal {L}}(w^T\phi ({\tilde{x}}), y) \end{aligned}$$

If we use second-order Taylor approximation at ${\mathbb {E}}_{{\tilde{x}}} [\phi ({\tilde{x}})]$, which is the centroid of the augmentation feature on the embedding space, then the prediction loss can be approximated as:

$$\begin{aligned} R_{\text {aug}} \approx \sum _{y} \underbrace{{\mathcal {L}}(w^{T} {\mathbb {E}}_{{\tilde{x}}}[\phi (x)], y)}_{(1)} + \underbrace{\frac{1}{2} {\mathbb {E}}_{{\tilde{x}}} [(w^{T} (\phi ({\tilde{x}})-{\mathbb {E}}_{{\tilde{x}}} [\phi ({\tilde{x}})]))^2 {\mathcal {L}}^{\prime \prime }(w^{T}{\mathbb {E}}_{{\tilde{x}}} [\phi ({\tilde{x}})],y)}_{(2)} \end{aligned}$$

The analysis reveals the augmentation training aims to: (1) encourage a small loss on the centroid of the generated feature, (2) indicates a smooth prediction on the new generated sample. Since ${\mathcal {L}}^{\prime \prime }(w^{T}{\mathbb {E}}_{{\tilde{x}}} [\phi ({\tilde{x}})],y)\le 1$ and $\phi$ is Lipschitz function, (2) can be further upper-bounded by:

$$\begin{aligned} \text {(2)} \le L^2_{\phi } \frac{\Vert w\Vert ^2_2}{4}\text {Var}({\tilde{x}}) \end{aligned}$$

Therefore, if the embedding function has a small Lipschitz constant, the second-order approximation of the augmentation loss can be controlled. Therefore, minimizing the prediction loss on the augmented data can be viewed as an implicit approach to encourage the Lipschitz representation.

## Experiments

In this section, we aim to empirically validate the effectiveness of the regularization term. And we want to address the following question: *Is the regularization term effective to generalize in the related unseen environments?*

### Choice of invariance criteria and loss

We evaluate the proposed regularization through typical invariance representation algorithms to verify the effectiveness of the regularization.

1. *Marginal Feature Alignment* In the marginal matching, we adopted the well-known Domain Adversarial Neural Network (i.e, DANN) (Ganin et al. 2016), which encourages ${\mathcal {S}}_1(z)=\dots ={\mathcal {S}}_T(z)$ through min-max optimization. Concretely, we introduce a domain discriminator $d:{\mathcal {Z}}\rightarrow \{1,\dots ,T\}$, such that
  $$\begin{aligned} \min _{\phi } \text {INV}(\phi ,{\mathcal {S}}_1,\dots ,{\mathcal {S}}_T) = \min _{\phi }\max _{d}\,\frac{1}{T} \sum ^{T}_{t=1} {\mathbb {E}}_{x_t \sim {\mathcal {S}}_t(x)} {\mathbf {1}}_{t} \log (d\circ \phi (x_t)), \end{aligned}$$
  where ${\mathbf {1}}_{t}$ is the one-hot vector. Intuitively, the discriminator tries to minimize the cross-entropy loss to differentiate the different sources, then the embedding function aims to learn an invariant representation to ensure ${\mathcal {S}}_1(z)=\dots ={\mathcal {S}}_T(z)$.
2. *Feature Conditional Invariance* We adopt the conditional-DANN (CDANN), which is adapted from Mirza and Osindero (2014) and Li et al. (2018). We introduce a conditional domain discriminator $d:{\mathcal {Z}}\times {\mathcal {Y}}\rightarrow \{1,\dots ,T\}$, such that:
  $$\begin{aligned} \min _{\phi } \text {INV}(\phi ,{\mathcal {S}}_1,\dots ,{\mathcal {S}}_T) = \min _{\phi }\max _{d}\,\frac{1}{T} \sum ^{T}_{t=1} {\mathbb {E}}_{(x_t,y_t) \sim {\mathcal {S}}_t(x,y)} {\mathbf {1}}_{t} \log \left( d\circ (\phi (x_t)\otimes y_t)\right) \end{aligned}$$
  Intuitively, the CDANN introduces a domain discriminator to differentiate different sources and their labels $z\otimes y$, then the representation learns the conditional invariant representation ${\mathcal {S}}_1(z|y)= \dots ={\mathcal {S}}_T(z|y)$.
3. *Label-Conditional Invariance* In this part, we adopted Invariant Risk Minimization (IRM), which is recently proposed by Arjovsky et al. (2019). Specifically, IRM adds a regularization term to encourage the ${\mathcal {S}}_1(y|z)= \dots ={\mathcal {S}}_T(y|z)$. They simply assume the predictor equals to 1 with
  $$\begin{aligned} \min _{\phi } \text {INV}(\phi ,{\mathcal {S}}_1,\dots ,{\mathcal {S}}_T) = \min _{\phi }\,\frac{1}{T} \sum ^{T}_{t=1} \Vert \nabla _{h|h=1} {\mathbb {E}}_{{\mathcal {S}}_t} {\mathcal {L}}(h\circ \phi (x_t),y_t)\Vert ^2 \end{aligned}$$
  As for the prediction loss ${\mathcal {L}}$, we adopted the conventional cross-entropy.^2^

### Dataset description and experimental setup

The experiment validation consists in evaluating toy and real-world datasets to verify the effectiveness of the regularization.

*ColorMNIST* (Arjovsky et al. 2019) Each MNIST image is either colored by red or green, in order to strongly correlate (but spuriously) with the class label. Thus the class label is strongly correlated with the color than with the digit configuration. The algorithm purely minimizing the training error will tend to exploit the false relation of the color, which will lead to a poor generalization of the unseen distribution with different color relations.

Following Arjovsky et al. (2019), the dataset is constructed as follows. (1) *Preliminary binary label*. We randomly select 5K samples from MNIST and construct preliminary binary label ${\tilde{y}}=0$ for digits 0-4 and ${\tilde{y}}=1$ for 5-9; (2) *Adding label noise*. We obtain the final label *y* by flipping ${\tilde{y}}$ with probability 0.25; (3) *Adding color as spurious feature*. We add the color to the gray-scale digit image by flipping *y* with probability $P_{{\mathcal {S}}}$ (i.e, coloring $y=1$ with red and $y=0$ with green by probability $1-P_{{\mathcal {S}}}$).

The ColorMNIST creates a *controllable* environment through assigning various $P_{{\mathcal {S}}}$, which enables us to evaluate the generalization performance under different unobserved environments.

*PACS* (Li et al. 2017) and *Office-Home* (Venkateswara et al. 2017) are real-world datasets with high-dimensional images. In PACS, the dataset consists four domains Photo (P), Art (A), Cartoon (C), Sketch (S) with 7 classes. In Office-Home, the dataset includes four domains Art (A), Clipart (C), Product (P) and Real World (R) with 65 classes.

*Experimental Setup* We use the standard domain generalization framework DomainBed (Gulrajani and Lopez-Paz 2021) to implement our algorithm. In ColorMNIST, we adopt the LeNet structure with three CNN layers as $\phi$ and three fc-layers as *h*. The mini-batch is set as 128 with Adam optimizer with $\lambda _0 = 1$, $\lambda _1\in [10^{-3},1]$. In PACS and Office-Home datasets, we adopt the pretrained ResNet-18 as $\phi$ and three fc-layers as *h*. We adopted training-domain validation set (Gulrajani and Lopez-Paz 2021) to search the best hyper-parameter configuration. Specifically, we set the batch size as 64 and $\lambda _0 \in [10^{-7},10^{-2}]$ and $\lambda _1 \in [10^{-5},1]$. We adopt the train-validation split approach (i.e, we randomly split the observed environment as training and validation sets and tune the best configuration on the validation set w.r.t. ${\mathcal {S}}$. We did not know the test environment during the tuning.) to search the best hyper-parameter. We run the experiments five times and report the average and std.

### Empirical results

The results are presented in Tables 1, 2 and 3. In all datasets and different invariance criteria, the regularization term suggests a consistent improvement (ranging from 1.2–6.2%). Specifically, the prediction improvement in the synthetic dataset (i.e. ColorMNIST) is significant, which verifies the effectiveness. Besides, in the real-world datasets such as Office-Home and PACS, the regularization suggests a consistent better performance.

**Table 1:** Table empirical results (Accuracy Per-Class on %, bold indicates a statistical significant result) on ColorMNIST

Method/Test Env	$P_{{\mathcal {S}}} = 0.1$	$P_{{\mathcal {S}}} = 0.2$	$P_{{\mathcal {S}}} = 0.9$	Average
ERM	60.2 ± 0.9	65.7 ± 0.6	26.8 ± 1.8	50.9
ERM+REG	**65.0** ± **1.9**	**69.4** ± **1.6**	**29.1** ± **1.3**	54.5
DANN	60.3 ± 2.3	66.2 ± 0.5	26.7 ± 2.5	51.1
DANN+REG	**68.2** ± **1.3**	**70.9** ± **1.7**	27.9 ± 2.1	55.7
CDANN	62.7 ± 1.9	66.7 ± 2.0	27.1 ± 3.2	52.2
CDANN+REG	**70.3** ± **0.5**	**72.2** ± **1.2**	**30.6** ± **1.7**	57.7
IRM	57.2 ± 1.7	63.3 ± 2.1	40.7 ± 10.5	53.7
IRM +REG	**61.9** ± **1.6**	**66.5** ± **3.3**	**51.2** ± **1.5**	59.9

We have three environments with different $P_{{\mathcal {S}}} = \{0.1, 0.2, 0.9\}$, which follows the experimental protocol of Arjovsky et al. (2019). In domain-generalization, we train on two environments and test on the untrained environment

**Table 2:** Empirical results (Accuracy Per-Class on %, bold indicates a statistical significant result) on PACS

Method/Test Env	Art	Cartoon	Sketch	Photo	Average
ERM	74.2 ± 1.2	71.8 ± 1.1	93.4 ± 0.9	71.4 ± 0.6	77.7
ERM+REG	**77.4** ± **1.4**	73.1 ± 0.7	**94.8** ± **0.8**	**73.5** ± **1.7**	79.7
DANN	77.3 ± 1.7	74.4 ± 1.5	93.3 ± 1.1	71.7 ± 2.5	79.2
DANN+REG	**81.1** ± **1.6**	**75.4** ± **0.7**	**94.8** ± **1.2**	**75.8** ± **1.1**	81.6
CDANN	79.6 ± 2.1	75.4 ± 1.8	93.8 ± 1.2	72.3 ± 1.1	80.3
CDANN+REG	**82.5** ± **0.5**	**78.1** ± **0.5**	**95.4** ± **0.8**	**77.0** ± **0.8**	83.3
IRM	69.0 ± 1.3	68.3 ± 1.7	88.7 ± 2.5	64.3± 1.2	72.6
IRM+REG	**73.7** ± **1.9**	**70.9** ± **2.5**	**92.1** ± **1.3**	**67.2** ± **2.0**	76.0

We have four environments Photo (P), Art (A), Cartoon (C) and Sketch (S). In domain-generalization, we train the model on three environments and test on the untrained environment

**Table 3:** Empirical results (Accuracy Per-Class on %, bold suggests a statistical significant result) on Office-Home

Method/Test Env	Art	Clipart	Product	Real-world	Average
ERM	46.8 ± 0.9	41.2 ± 0.8	64.5 ± 1.1	66.1 ± 0.7	54.7
ERM+REG	**48.7** ± **0.9**	42.1 ± 1.0	65.5 ± 0.7	67.1 ± 0.6	55.9
DANN	48.0 ± 0.8	44.4 ± 0.9	65.7 ± 1.2	66.5 ± 0.8	56.1
DANN+REG	**50.5** ± **1.1**	**46.0** ± **0.8**	**68.0** ± **0.8**	**68.5** ± **0.9**	58.3
CDANN	48.6 ± 1.1	44.7 ± 0.7	65.6 ± 1.1	66.3 ± 0.8	56.3
CDANN+REG	**52.0** ± **1.3**	**47.2** ± **0.7**	**67.9** ± **0.8**	**69.4** ± **1.0**	59.1
IRM	47.2 ± 0.7	42.3 ± 1.9	63.4 ± 1.5	65.3 ± 2.2	54.6
IRM+REG	**49.1** ± **1.2**	43.8 ± 1.3	**66.1** ± **1.2**	**68.4** ± **1.8**	56.9

We have four environments Art (A), Clipart (C), Product (P) and Real-world (R). In the domain-generalization, we train the model on three environments and test on the untrained environment

### Analysis

We further conduct various analysis to understand the properties and role of regularization.

*Influence of regularization*

For a better understanding the influence of regularization, we gradually change $\lambda _1$ and evaluate the test environment prediction error. The empirical results are consistent with our theoretical analysis: for a small regularization, the prediction performance can be improved. However, a strong regularization (over smoothing) on the representation learning can be harmful, with a clear performance drop.

**Fig. 4:**
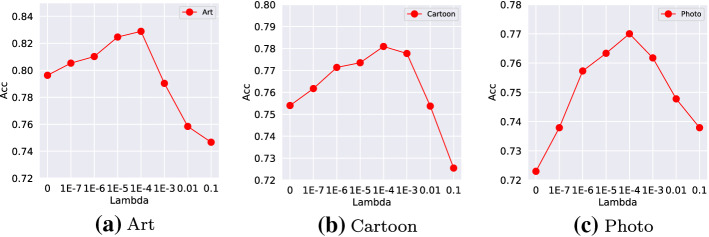
Influence of regularization in PACS dataset in CDANN. We gradually change the weights of regularization (i.e, different $\lambda _1$). The accuracy first increases with a larger $\lambda _1$, then the accuracy drops due to a over-regularization on the representation

**Fig. 5:**
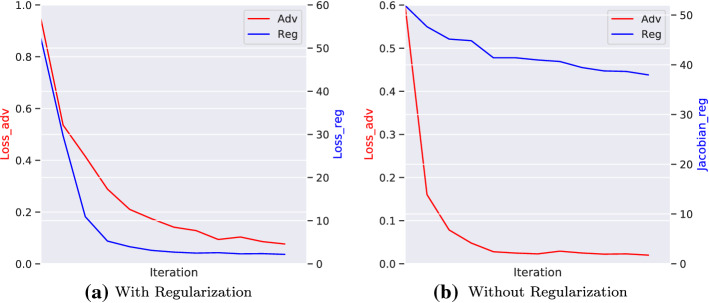
Loss Evolution in Office-Home dataset (Training Environments: Clipart, Product, Real-World) in CDANN. Left: The evolution of adversarial loss and regularization term if we adopt the regularization loss. Right: The evolution of adversarial loss and regularization term (Norm of Jacobian matrix) *without* adopting regularization loss. The results reveal that without explicit regularization loss, the norm of Jacobian matrix can gradually (but slowly) diminishes. In contrast, adding an explicit term can explicit ensure a small Lipschitz constant

*Evolution of Training* We additionally visualize the evolution of adversarial loss and the norm of Jacobian matrix in two training modes: conditional alignment with (w.) and without (w.o.) regularization. Clearly, training without explicit regularization leads to a relative large norm of Jacobian matrix. In the optimization procedure, the norm of Jacobian matrix gradually but slowly diminishes, which is possibly caused by the implicit regularization through stochastic gradient descent (SGD) based approach (Roberts et al. 2021). Therefore, adding an explicit regularization term can induce a better generalization (Figs. 4, 5).

**Fig. 6:**
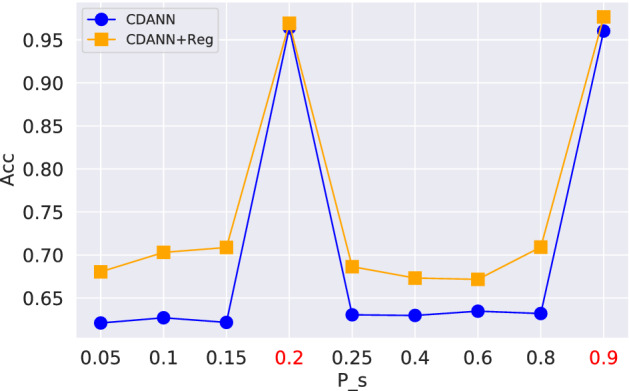
Generalization on different test environments. The observed environments are $P_{{\mathcal {S}}}=\{{0.2},{0.9}\}$ with high prediction performance. However, in the generalization on other test environments with different $P_{{\mathcal {S}}}$, the regularization term can consistently improve the prediction performance

*Generalization in controllable environment* In order to better understand the behavior in domain generalization, we create the controllable environments in ColorMNIST. Specifically, we fix the observed environments $P_{{\mathcal {S}}}=\{0.2, 0.9\}$ and test on various environments with different $P_{{\mathcal {T}}}=\{0.05,\dots ,0.85\}$, shown in Fig. 6. In the observed environments $P_{{\mathcal {S}}}=\{0.2, 0.9\}$, both approaches achieve high prediction accuracy with larger than 95%. However, their generalization behaviors in other environments are quite different: adding an regularization term consistently improves the performance in out-of-distribution prediction through 3–5%.

## Conclusion

In this paper, we analyzed the representation-learning based domain generalization. Concretely, we highlight the importance of regularizing the representation function. Then we theoretically demonstrate the benefits of regularization, as the key role to control the prediction error in the unseen test environment. In practice, we evaluate the Jacobian matrix regularization on various invariance criteria and datasets, which suggests the benefits of regularization. In the future work, we aim to explore the relation between meta-learning based domain generalization (Blanchard et al. 2011) or other types of discrepancy such as deep MMD (Liu et al. 2020).

## Data Availability

Not applicable.

## Appendix: Proofs

### *Proof of Proposition*1

The prediction error in the test environment can be expressed as:

$$\begin{aligned} {\mathrm {BER}}_T(h,{\varPhi })&= \frac{1}{|{\mathcal {Y}}|}\sum _{y=1}^{{\mathcal {Y}}} \int _{z} T(z|Y=y){\mathcal {L}}(h(z),y) \\&\le \frac{1}{|{\mathcal {Y}}|}\sum _{y=1}^{{\mathcal {Y}}} \left[ {\mathbb {E}}_{z \sim {\mathcal {S}}^{\star }(z|Y=y)}{\mathcal {L}}(h(z),y) + d_{{\mathrm {TV}}}({\mathcal {S}}^{\star }(z|Y=y)\Vert {\mathcal {T}}(z|Y=y)) \right] \end{aligned}$$

Where ${\mathcal {S}}^{\star }$ is the nearest source environment that is the most similar to the test environment (i.e. in the raw feature space, $d_{{\mathrm {TV}}}({\mathcal {S}}^{\star }(x|Y=y)\Vert {\mathcal {T}}(x|Y=y))\le \epsilon$) We have the following upper since the prediction loss in upper bounded by 1 and the property of TV distance (Polyanskiy and Wu 2019, Remark 3.1).

We analyzed the first term, since ${\mathcal {S}}^{\star }$ is unknown source during the training, then we can upper bound through all the sources, i.e, $\forall t\in \{1,\dots ,T\}$, we have:

$$\begin{aligned} {\mathbb {E}}_{z \sim {\mathcal {S}}^{\star }(z|Y=y)}{\mathcal {L}}(h(z),y) \le \frac{1}{T}\sum _{t=1}^T {\mathbb {E}}_{z \sim {\mathcal {S}}_{t}(z|Y=y)}{\mathcal {L}}(h(z),y) + \frac{1}{T}\sum _{t=1}^T d_{{\mathrm {TV}}}({\mathcal {S}}^{\star }(z|Y=y)\Vert {\mathcal {S}}_t(z|Y=y)) \end{aligned}$$

The proof of the above inequality is analogous to the first inequality and derived by the property of TV distance. Concretely, we use the inequality *T*-times and then derive the average upper bound.

Since we adopt the feature conditional invariance criteria, then we have $d_{{\mathrm {TV}}}({\mathcal {S}}^{\star }(z|Y=y)\Vert {\mathcal {S}}_t(z|Y=y))\le \kappa$. This inequality holds since in training we have enforced a small conditional invariance among all sources. Then this term can be upper bounded by:

$$\begin{aligned} {\mathbb {E}}_{z \sim {\mathcal {S}}^{\star }(z|Y=y)}{\mathcal {L}}(h(z),y) \le \frac{1}{T}\sum _{t=1}^T {\mathbb {E}}_{z \sim {\mathcal {S}}_{t}(z|Y=y)}{\mathcal {L}}(h(z),y) + \kappa \end{aligned}$$

Next, we upper bound the second term through introducing the strong data-processing inequality (Polyanskiy and Wu 2019). The strong data-processing suggests a tighter bound w.r.t. the conventional data-processing inequality. Specifically, it reveals the decay rate of information loss, characterized by the Dobrushin coefficient.

*Strong data-processing inequality* For distributions $P_0,P_1$ defined on ${\mathcal {X}}$ and a channel *Q* from space ${\mathcal {X}}$ to space ${\mathcal {Z}}$, define a marginal distribution $M_0(z) = \int Q(z|x)P_0(x)dx$. The channel *Q* satisfies a strong data processing inequality with constant $\alpha \le 1$ for the given *f*-divergence.

$$\begin{aligned} D_f(M_0\Vert M_1) \le \alpha _f D_f(P_0\Vert P_1) \end{aligned}$$

Where $\alpha$ is a constant defined with $\alpha _f(Q) = \sup _{P_0 \ne P_1} \frac{ D_f(M_0\Vert M_1)}{D_f(P_0\Vert P_1)}$. For any convex *f* divergence, we have:

$$\begin{aligned} \alpha _f(Q)\le \alpha _{\text {TV}}(Q) \end{aligned}$$

Where $\alpha _{\text {TV}}(Q)$ is the *Dobrushin coefficient*, which is equivalent as:

$$\begin{aligned} \alpha _{\text {TV}}(Q) :=\sup _{x,x^{\prime }}d_{\text {TV}}(Q(\cdot |x)\Vert Q(\cdot |x^{\prime })) \end{aligned}$$

In the context of representation learning, the embedding distribution ${\varPhi }$ can be viewed as the information channel, and we denote distributions $P_0$ and $P_1$ as ${\mathcal {S}}^{\star }(x|Y=y)$ and ${\mathcal {T}}(x|Y=y)$. The conditional distributions defined on the latent space are ${\mathcal {S}}^{\star }(z|Y=y) = \int {\varPhi }(z|x){\mathcal {S}}^{\star }(x|Y=y)dx$, ${\mathcal {T}}(z|Y=y) = \int {\varPhi }(z|x){\mathcal {T}}(x|Y=y)dx$. Then we have:

$$\begin{aligned} d_{\text {TV}}({\mathcal {S}}^{\star }(z|y)\Vert {\mathcal {T}}(z|y)) \le \alpha _{\text {TV}}({\varPhi }) d_{{\mathrm {TV}}}({\mathcal {S}}^{\star }(z|Y=y)\Vert {\mathcal {T}}(z|Y=y)) \le \alpha _{\text {TV}}({\varPhi })\epsilon \end{aligned}$$

Plugging in all the elements, we have the upper bound:

$$\begin{aligned} {\mathrm {BER}}_T(h,{\varPhi }) \le \frac{1}{|{\mathcal {Y}}|}\sum _{y=1}^{{\mathcal {Y}}}(\frac{1}{T}\sum _{t=1}^T {\mathbb {E}}_{z \sim {\mathcal {S}}_{t}(z|Y=y)}{\mathcal {L}}(h(z),y) + \kappa + \alpha _{\text {TV}}({\varPhi })\epsilon ) \end{aligned}$$

Rearranging the results, we have:

$$\begin{aligned} \text {BER}_{{\mathcal {T}}}(h,{\varPhi }) \le \frac{1}{T} \sum _{t=1}^T \text {BER}_{{\mathcal {S}}_t}(h,{\varPhi }) + \kappa + \alpha _{{\mathrm {TV}}}({\varPhi })\epsilon \end{aligned}$$

$$\square$$

### *Proof of Lemma*1

We first prove the relation with feature conditional invariance and marginal invariance.

*Relation with marginal invariance* According to the definition, we have:

$$\begin{aligned} {\mathbb {E}}_{z\sim {\varOmega }^{\star }}|{\mathcal {S}}_i(z)-{\mathcal {S}}_j(z)|&= {\mathbb {E}}_{z\sim {\varOmega }^{\star }}|\sum _{y}{\mathcal {S}}_i(y){\mathcal {S}}_i(z|y)-\sum _{y}{\mathcal {S}}_j(y){\mathcal {S}}_j(z|y)|\\&= \frac{1}{|{\mathcal {Y}}|}{\mathbb {E}}_{z\sim {\varOmega }^{\star }} |\sum _{y}({\mathcal {S}}_i(z|y)-{\mathcal {S}}_j(z|y))| \\&\le \frac{1}{|{\mathcal {Y}}|} \sum _{y} {\mathbb {E}}_{z\sim {\varOmega }^{\star }}|{\mathcal {S}}_i(z|y)-{\mathcal {S}}_j(z|y)|\\&= \frac{1}{|{\mathcal {Y}}|} \sum _{y} d_{\text {TV}}({\mathcal {S}}_i(z|y)\Vert {\mathcal {S}}_j(z|y)) \le \kappa \end{aligned}$$

*Relation with label conditional invariance* According to the definition, we have:

$$\begin{aligned} {\mathbb {E}}_{z\sim {\varOmega }^{\star }} |{\mathcal {S}}_i(y|z)-{\mathcal {S}}_j(y|z)|&= \int _{z\sim {\varOmega }^{\star }} |\frac{{\mathcal {S}}_i(z|y){\mathcal {S}}_i(y)}{\sum _{y}{\mathcal {S}}_i(y){\mathcal {S}}_i(z|y)} - \frac{{\mathcal {S}}_j(z|y){\mathcal {S}}_j(y)}{\sum _{y}{\mathcal {S}}_j(y){\mathcal {S}}_j(z|y)}|\\&= \int _{z\sim {\varOmega }^{\star }} |\frac{{\mathcal {S}}_i(z|y)}{\sum _{y}{\mathcal {S}}_i(z|y)} - \frac{{\mathcal {S}}_j(z|y)}{\sum _{y}{\mathcal {S}}_j(z|y)}|\\&\le \int _{z\sim {\varOmega }^{\star }} |\frac{{\mathcal {S}}_i(z|y)}{\sum _{y} {\mathcal {S}}_i(z|y)} - \frac{{\mathcal {S}}_i(z|y)}{\sum _{y} {\mathcal {S}}_j(z|y)}| + |\frac{{\mathcal {S}}_i(z|y)}{\sum _{y} {\mathcal {S}}_j(z|y)} - \frac{{\mathcal {S}}_j(z|y)}{\sum _{y} {\mathcal {S}}_j(z|y)} | \end{aligned}$$

We start to upper bound this two terms. For the first term, we have:

$$\begin{aligned} \int _{z\sim {\varOmega }^{\star }} |\frac{{\mathcal {S}}_i(z|y)}{\sum _{y} {\mathcal {S}}_i(z|y)} - \frac{{\mathcal {S}}_i(z|y)}{\sum _{y} {\mathcal {S}}_j(z|y)}|&= \int _{z\sim {\varOmega }^{\star }} {\mathcal {S}}_i(z|y)\frac{|\sum _y [{\mathcal {S}}_j (z|y) - {\mathcal {S}}_i (z|y)]|}{[\sum _{y}{\mathcal {S}}_i(z|y)][\sum _{y}{\mathcal {S}}_j(z|y)]}\\&= \int _{z\sim {\varOmega }^{\star }} \frac{{\mathcal {S}}_i(z|y)}{\sum _{y}{\mathcal {S}}_i(z|y)} \frac{\sum _{y}|{\mathcal {S}}_j (z|y) - {\mathcal {S}}_i (z|y)|}{\sum _{y}{\mathcal {S}}_j(z|y)}\\&\le \int _{z\sim {\varOmega }^{\star }} \frac{\sum _{y}|{\mathcal {S}}_j (z|y) - {\mathcal {S}}_i (z|y)|}{\sum _{y}{\mathcal {S}}_j(z|y)} \\&\le C_1 \sum _{y} \int _{z} |{\mathcal {S}}_j (z|y) - {\mathcal {S}}_i (z|y)| \\&\le C_1 |{\mathcal {Y}}| \kappa \end{aligned}$$

Where $C_1 = \frac{1}{\inf _{z\in {\varOmega }^{\star }}\sum _{y} {\mathcal {S}}_j(z|y)}$ and we can verify $C_1>0$ since ${\varOmega }^{\star }$ is the intersection region with non-zero measure.

Then we bound the second term:

$$\begin{aligned} \int _{z} |\frac{{\mathcal {S}}_i(z|y)}{\sum _{y} {\mathcal {S}}_j(z|y)} - \frac{{\mathcal {S}}_j(z|y)}{\sum _{y} {\mathcal {S}}_j(z|y)}| \le \frac{1}{\inf _{z\in {\varOmega }^{\star }}\sum _{y} {\mathcal {S}}_j(z|y)} \int _{z}|{\mathcal {S}}_i(z|y)-{\mathcal {S}}_j(z|y)| = C_1 \kappa \end{aligned}$$

Combining all results, we have:

$$\begin{aligned} {\mathbb {E}}_{z\sim {\varOmega }^{\star }} |{\mathcal {S}}_i(y|z)-{\mathcal {S}}_j(y|z)| \le C_1(1+|{\mathcal {Y}}|)\kappa = C^{+}\kappa \end{aligned}$$

Where $C^{+}= C_1(1+|{\mathcal {Y}}|)$ is a positive constant. $\square$

### *Proof of Lemma* 2

Since we approximate the ${\varPhi }$ as a multi-dimensional Gaussian distribution, then the Dobrushin Coefficient can be computed as:

$$\begin{aligned} \sup _{x,x^{\prime }}\,d_{{\mathrm {TV}}}({\mathcal {N}}(\phi (x),\sigma ^2 {\mathbf {I}}_{d})\Vert {\mathcal {N}}(\phi (x^{\prime }),\sigma ^2 {\mathbf {I}}_{d})) \end{aligned}$$

Since the TV distance of multidimensional Gaussian is infeasible to compute, then according to Devroye et al. (2018), the upper bound of TV distance between two high-dimensional Gaussian distributions is:

$$\begin{aligned} d_{{\mathrm {TV}}}({\mathcal {N}}(\phi (x),\sigma ^2 {\mathbf {I}}_{d})\Vert {\mathcal {N}}(\phi (x^{\prime }),\sigma ^2 {\mathbf {I}}_{d})) \le \sqrt{2} d_{H}({\mathcal {N}}(\phi (x),\sigma ^2 {\mathbf {I}}_{d})\Vert {\mathcal {N}}(\phi (x^{\prime }),\sigma ^2 {\mathbf {I}}_{d})) \end{aligned}$$

Where $d_{H}$ is the Hellinger distance, which has the closed form of between two Gaussian distributions with

$$\begin{aligned} d_{H}({\mathcal {N}}(\phi (x),\sigma ^2 {\mathbf {I}}_{d}), {\mathcal {N}}(\phi (x^{\prime }),\sigma ^2 {\mathbf {I}}_{d})) = \left( 1- \exp (-\frac{1}{8\sigma ^2 d} [\phi (x)-\phi (x^{\prime })]^{T} [\phi (x)-\phi (x^{\prime })])\right) ^{1/2} \end{aligned}$$

Then the TV distance can be upper bounded as:

$$\begin{aligned} \alpha _{{\mathrm {TV}}}({\varPhi }) \le \sup _{x,x^{\prime }\in {\mathcal {X}}} \sqrt{2}\left( 1- \exp (-\frac{1}{8d \sigma ^2} \Vert \phi (x)-\phi (x^{\prime })\Vert ^2 )\right) ^{1/2} \end{aligned}$$

We assume $\phi$ is $L_{\phi }$ Lipschitz such that w.r.t. *x*,

$$\begin{aligned} \Vert \phi (x)-\phi (x^{\prime })\Vert \le L_{\phi } \Vert x-x^{\prime } \Vert _2 \end{aligned}$$

and the $d_{\max } = \sup _{x,x^{\prime }}\Vert x-x^{\prime } \Vert _2$. Then we have:

$$\begin{aligned} \alpha _{{\mathrm {TV}}}({\varPhi }) \le \sqrt{2}\left( 1- \exp (-\frac{d^2_{\max }}{8d \sigma ^2} L^2_{\phi } )\right) ^{1/2} \end{aligned}$$

$$\square$$

## Relation with data-augmentation

In this part, we propose a simple proof to show the role of data-augmentation, which also aims at regularizing the representation.

We suppose a differentiable embedding function $\phi :{\mathcal {X}}\rightarrow {\mathcal {Z}}$, the loss as logistic function ${\mathcal {L}}({\hat{y}},y) = \log (1+\exp (-{\hat{y}}y))$ and the predictor as a linear function *w*, binary classification with balanced label distribution. Then the objective function can be written as:

$$\begin{aligned} {\mathcal {G}}(w) = {\mathbb {E}}_{{\tilde{x}}}\,{\mathcal {L}}(w^{T}\phi ({\tilde{x}}),y) \end{aligned}$$

Where ${\tilde{x}}=\text {INP}(x_1,\dots ,x_T), x_1\sim {\mathcal {S}}_1(x|Y=y), \dots , x_T\sim {\mathcal {S}}_T(x|Y=y)$ is any interpolation function of samples from multiple environments. We also suppose the data augmentation aims at improving the local property of the representation $\phi$. Then by using first-order Taylor expansion at the local representation $\phi _0$, we have:

$$\begin{aligned} {\mathcal {G}}_1(w) = {\mathcal {L}}(w^{T}\phi _0, y) + {\mathbb {E}}_{{\tilde{x}}} (\phi _0 - \phi ({\tilde{x}})) {\mathcal {L}}^{\prime }(w^{T}\phi _0,y) \end{aligned}$$

If we take $\phi _0(x) = {\mathbb {E}}_{{\tilde{x}}} [\phi ({\tilde{x}})]$, then the second term vanish, then the first order approximation can be expressed as:

$$\begin{aligned} {\mathcal {G}}_1(w) = {\mathcal {L}}(w^{T} {\mathbb {E}}_{{\tilde{x}}}[\phi (x)], y) \end{aligned}$$

Then we compute the second-order approximation at point $\phi _0$, then we have

$$\begin{aligned} {\mathcal {G}}_2(w) = \frac{1}{2} {\mathbb {E}}_{{\tilde{x}}} [(w^{T} (\phi ({\tilde{x}})-{\mathbb {E}}_{{\tilde{x}}} [\phi ({\tilde{x}})]))^2 {\mathcal {L}}^{\prime \prime }(w^{T}{\mathbb {E}}_{{\tilde{x}}} [\phi ({\tilde{x}})],y) \end{aligned}$$

We can further compute that if ${\mathcal {L}}$ is logistic loss, the second-derivative is independent of label *y* and the second derivative is bounded by 1. Then we have

$$\begin{aligned} {\mathcal {G}}_2(w) \le \frac{1}{2} \text {Var}_{{\tilde{x}}} (w^{T}\phi ({\tilde{x}}))^2 \end{aligned}$$

*Relation with regularization term* If the embedding function is $L_{\phi }$ Lipschitz then the function $w^{T}\phi ({\tilde{x}})$ is also $L_{\phi }\Vert w\Vert _2$-Lipschitz through:

$$\begin{aligned} |w^{T}\phi ({\tilde{x}}_1) - w^{T}\phi ({\tilde{x}}_2)| \le \Vert w\Vert _2 \Vert \phi ({\tilde{x}}_1)-\phi ({\tilde{x}}_2)\Vert _2 \le L_{\phi }\Vert w\Vert _2 \Vert {\tilde{x}}_1-{\tilde{x}}_2\Vert \end{aligned}$$

Then we have the upper bound of ${\mathcal {G}}_2 \le L^2_{\phi } \frac{\Vert w\Vert ^2_2}{4}\text {Var}({\tilde{x}})$. Therefore, the minimize the loss on the augmented data set can be viewed as an implicit optimization to enforce a small prediction variance, where the Lipschitz representation function $\phi$ is one sufficient condition to realize it.

### *Proof*

We can compute the second derivative of ${\mathcal {L}}({\hat{y}},y) = \log (1+\exp (-{\hat{y}}y))$ w.r.t. ${\hat{y}}$:

$$\begin{aligned} \frac{\partial ^2 {\mathcal {L}}({\hat{y}},y)}{\partial {\hat{y}}^2} = \frac{y^2\exp (y{\hat{y}})}{(1+\exp (y{\hat{y}}))^2} \end{aligned}$$

Since *y* is binary with possible values $y=\{-1,+1\}$, then we have $y^2=1$, the second-derivative is independent of *y* with $\frac{\partial ^2 {\mathcal {L}}({\hat{y}},y=1)}{\partial {\hat{y}}^2} = \frac{\partial ^2 {\mathcal {L}}({\hat{y}},y=-1)}{\partial {\hat{y}}^2} = \frac{\exp ({\hat{y}})}{(1+\exp ({\hat{y}}))^2} \le 1$ $\square$

## Appendix: The network structure

Shown Tables 4 and 5.

**Table 4:** Network structure in digits recognition.

Feature extractor $\phi$	
conv 1	$3\times 3 \times 64$
conv 2	$3\times 3 \times 128$
conv 3	$3\times 3 \times 256$
Classifier *h*	
fc 1	$\star \times 512$
fc 2	$512 \times 100$
fc 3	$100 \times 2$
Domain discriminator *d*	
fc 1	$\star \times 256$
fc 2	$256 \times \text {environment}\_\text {number}$

**Table 5:** Network structure in PACS and Office-Home

Feature extractor $\phi$	
ResNet18 pre-trained network	
Classifier *h*	
fc 1	$\star \times 256$
fc 2	$256 \times 256$
fc 3	$256 \times \text {class}\_\text {number}$
Domain discriminator *d*	
fc 1	$\star \times 256$
fc 2	$256 \times 256$
fc 3	$256 \times {\text{environment\_number}}$

## Footnotes

1 It is worth mentioning that Lipschitz constant is one sufficient condition (or upper bound) to control Dobrushin Coefficient. It can be further incorporated with data-dependent regularization to better control $\alpha _{\text {TV}}({\varPhi })$.

2 It should be pointed out the cross entropy loss is generally not bounded. However, the empirical results suggest its effectiveness in practice.
